# Changes in the Localization of Polyamine Spermidine in the Rat Retina with Age

**DOI:** 10.3390/biomedicines11041008

**Published:** 2023-03-24

**Authors:** David S. Ríos, Christian J. Malpica-Nieves, Amanda Díaz-García, Misty J. Eaton, Serguei N. Skatchkov

**Affiliations:** 1 College of Science and Health Professions, Universidad Central de Bayamón, Bayamón, PR 00960, USA; 2 Department of Biochemistry, Universidad Central del Caribe, Bayamón, PR 00956, USA; 3 Department of Physiology, Universidad Central del Caribe, Bayamón, PR 00956, USA

**Keywords:** polyamines, spermidine, nervous system, retina, glial cells, neurons, glial cell compartments

## Abstract

Polyamines (PAs) in the nervous system has a key role in regeneration and aging. Therefore, we investigated age-related changes in the expression of PA spermidine (SPD) in the rat retina. Fluorescent immunocytochemistry was used to evaluate the accumulation of SPD in retinae from rats of postnatal days 3, 21, and 120. Glial cells were identified using glutamine synthetase (GS), whereas DAPI, a marker of cell nuclei, was used to differentiate between retinal layers. SPD localization in the retina was strikingly different between neonates and adults. In the neonatal retina (postnatal day 3-P3), SPD is strongly expressed in practically all cell types, including radial glia and neurons. SPD staining showed strong co-localization with the glial marker GS in Müller Cells (MCs) in the outer neuroblast layer. In the weaning period (postnatal day 21-P21), the SPD label was strongly expressed in all MCs, but not in neurons. In early adulthood (postnatal day 120-P120), SPD was localized in MCs only and was co-localized with the glial marker GS. A decline in the expression of PAs in neurons was observed with age while glial cells accumulated SPD after the differentiation stage (P21) and during aging in MC cellular endfoot compartments.

## 1. Introduction

PAs are polycations composed of flexible carbon chains with positively charged amino groups that bind negatively charged molecules such as DNA, RNA, and acid proteins [1]. Spermidine (SPD) is synthesized from the precursor putrescine (PUT), by the enzyme S-adenosyl-L-methionine decarboxylase (SAM dc) [2]. PUT levels are established by the regulated synthetic enzyme ornithine decarboxylase (ODC), with L-ornithine [3,4,5] and L-arginine [6] as precursors of PUT. Modified ornithine (alpha-difluoromethylornithine (DFMO)) is a blocker of ODC [5]. In the classical synthetic pathway, the enzyme ODC uses L-ornithine as a substrate and synthesizes PUT by decarboxylation. Alternatively, PAs may be synthesized via arginine or agmatine pathways [6,7,8]. After PUT production, the synthetic enzyme spermidine synthase consequently converts PUT to SPD.

PAs (including the diamine PUT and the triamine SPD) are widespread in living organisms [9]. PAs are accumulated in actively proliferating glial cells [10], and are involved in a variety of fundamental cellular processes, such as stabilization of DNA, transcription, RNA modification, protein synthesis and the modulation of enzyme activity, cell proliferation, differentiation, migration, and apoptosis [11,12,13]. In the CNS, PAs are important for glial–neuronal communication because they regulate numerous receptors and channels expressed in glia and neurons, including ionotropic glutamate receptors (NMDA [14,15] and AMPA/kainate [16,17]), the inward rectifier (Kir) K^+^ channels, specifically glial Kir4.1 [18,19,20], calcium-activated chloride channels [21], TRP channels [22,23], ASIC channels [24,25] and other receptors and channels [26]. Since PAs affect many neuronal and glial receptors, these molecules are key elements for normal brain and retinal function.

In the retina, PAs are essential for numerous processes [19,27,28]. They are gating molecules for inwardly rectifying K^+^ channels [27,29] and in Müller glial retinal cells, PAs are involved in important glial cell functions such as the clearance of excess extracellular K^+^ ions [19,27,30]. Also of interest is the report that PAs may regulate dark adaptation through their inhibition of the cyclic nucleotide-gated calcium channel in photoreceptors [31]. Amongst all its roles, SPD also acts as an endogenous free radical scavenger that inhibits the action of reactive oxygen species [8,13,26]. Furthermore, daily intake of SPD was found to reduce ganglion cell death and enhance optic nerve regeneration following an optic nerve injury [28]. This is critical since PA content in the retina and brain has been found to decrease with age [32].

The PA spermine (SPM) is localized predominantly in glia, not in neurons, in the brain and retina [8,19,26,27,33,34]. In the tiger salamander retina, the most abundant PAs are PUT, SPD, and SPM and the staining of PAs in tiger salamanders shows labeling in ganglion and amacrine cells [35]. PAs were also observed in adult rabbit retina [27,36], goldfish [32], and human retina [27]. In rabbit retina, SPM immunostaining was also found in the photoreceptors (rods and cones), where the concentration of PAs has been found to decrease with age [36]. Furthermore, cone loss occurs if PA synthesis and ODC are blocked by DFMO [36]. Biedermann et al. [27] clearly showed specific labeling of PAs in Müller glial cells from guinea pig and porcine retina and their important role in the functioning of K^+^ channels [19]. In rat retinas, PA content and biosynthesis were measured in fractioned rat retinas [3] and it was found that SPD and SPM content also declined after iodoacetate (a gliotoxin and photoreceptor cell toxin). A similar decline was observed after another gliotoxin, fluoroacetate, in the brain [37]. In organs, and specifically, in the brain [38] and retina [36], PA content decreases with age. The purpose of this study was to (1) determine the detailed localization of the SPD in rat retina and (2) examine possible changes in expression patterns with aging.

## 2. Materials and Methods

### 2.1. Animals and Tissues

Experiments were carried out under IACUC approval and in accordance with the ARVO Statement for the Use of Animals in Ophthalmic and Vision Research. Eyes were obtained from Sprague Dawley rats (postnatal days 3, 21, and 120) that were housed in a standard cage in a 12 h light–dark cycle room and had access to food (standard rat chow) and water freely. Rats were decapitated and eyes were rapidly enucleated and then processed for immunohistochemistry.

### 2.2. Immunohistochemistry

Following enucleation, eyes were fixed in a two-step process using two different solutions. The eyes were first fixed for 45 min in a solution consisting of 4% paraformaldehyde (Sigma-Aldrich, St. Louis, MO, USA, CAT#P6148) or 4% paraformaldehyde with 0.2% picric acid (Sigma-Aldrich, St. Louis, MO, USA, CAT#197378) and 0.05% glutaraldehyde (Sigma-Aldrich, St. Louis, MO, USA, CAT#G7651) in phosphate-buffered solution 0.1 M (PBS: NaCl 136.9 mM, KCl 2.7 mM, Na_2_ HPO_4_ 10.1 mM, KH_2_PO_4_ 1.8 mM with pH 7.4). Eyes were punctured with a 25 G needle in the ora serrata and fixed for an additional 20 min with a fresh fixative solution which consisted of 4% paraformaldehyde with 0.2% picric acid and 0.05% glutaraldehyde as described in [19,27,33]. Eyes were washed 3 times with PBS 0.1 M before separating the retina in a cold PBS solution using a stereoscopic microscope (Fisher Stereomaster, Waltham, MA, USA, CAT# FW00-20-1613). Fixed retinae were embedded in 4% Agarose (Gibco BRL, Waltham, MA, USA, CAT#15510-019) in PBS 0.1 M. A Leica VT 1000 S Vibratome (Leica, Germany) was used to obtain 20 μm retinal sections.

Samples were moved to a 24-well plate and permeabilized for 20 min with 1% DMSO (MP Biomedicals, Santa Ana, CA, USA, CAT#02196055,), 0.3% Triton X-100 (Sigma-Aldrich, St. Louis, MO, USA, CAT#T9284) in a PBS 0.1 M solution. The permeabilization solution was removed and replaced with a blocking solution containing: 2% bovine serum albumin (BSA; Sigma-Aldrich, St. Louis, MO, USA, CAT#A4503), and either 5% normal horse serum (Vector, Burlingame, CA, USA, CAT#S-2000) or 5% normal goat serum (Vector, Burlingame, CA, USA, CAT#S-1000) depending on the secondary antibody, 1% DMSO (MP Biomedicals, Santa Ana, CA, USA, CAT#196055) and 0.3% Triton X-100 (Sigma-Aldrich, St. Louis, MO, USA, CAT#T9284) in PBS 0.1 M for one hour. The blocking solution was replaced with a fresh solution containing primary antibodies and left shaking overnight at 4 °C. A rabbit anti-spermidine antibody (Abcam, Cambridge, UK, CAT#ab7318; 1/100,) was used to determine SPD localization (Note: the anti-spermidine antibody has primary reactivity for SPD and cross-reactivity with SPM but zero reactivity with PUT (reported by the Abcam company)). All samples were double labeled with glutamine synthetase (Millipore-Sigma, Massachusetts, USA, CAT#MABN1182; 1/250), a specific marker of glial cells. The primary antibody aliquot was removed and the permeabilization solution was used to wash the samples three times for 10 min while shaking. Then, green fluorescence anti-rabbit FITC (Vector, Burlingame, CA, USA, CAT#Fl-1000, 1:200) and red fluorescence anti-mouse Texas Red (Vector, Burlingame, CA, USA, CAT#Tl-2000; 1:200) secondary antibodies were diluted in the permeabilization solution and added to the samples. The sections were covered from light and incubated for two hours at 4 °C while shaking. After which, samples were washed three times for 10 min with PBS 0.1 M and once with distilled water. Tissue was mounted on slides, left to dry for 5 min, and Fluoroshield with DAPI (Sigma-Aldrich, St. Louis, MO, USA, CAT#F6057) or Hard Set Vectashield with the nuclear stain DAPI (Vector, Burlingame, CA, USA, CAT#H-1500) was added before sealing with coverslips. In preliminary experiments, non-specific staining by the secondary antibody was discarded by omitting the primary antibody from the reaction.

### 2.3. Confocal Microscopy

Confocal images were acquired using an Olympus BX60 microscope (Olympus, Tokyo, Japan) outfitted with an Olympus FV1000 confocal laser scanning system. Images were taken using 40× magnification. To ensure veracity, experiments were performed in triplicate. Image processing was performed using the Fluoview program, Image J (NIH, Bethesda, MD, USA), and Adobe Photoshop (Adobe Inc., San Jose, CA, USA).

### 2.4. Semi-Quantitative Analysis of Staining Intensity

Merged images obtained from confocal microscopy were analyzed using Image J software (version 2.1.0/1.53c). We measured 5 fluorescence spots in three different regions of the retina, (i) the endfoot area, (ii) the inner nuclear layer, and (iii) the outer nuclear layer in every image taken from retinal samples (n = 3). The fluorescence in each spot was measured and the mean of the spot fluorescence was imported to PRISM (Version 9.4.1 (458), GraphPad Software, San Diego, CA, USA) for statistical analysis.

### 2.5. Data Analysis and Statistics

Two-way ANOVA with multiple comparisons (Tukey’s multiple comparison test) was used to compare the mean fluorescence of the samples. Statistical difference was established to be *p* values lower than 0.05 with a 95% confidence interval.

## 3. Results

### 3.1. Immunohistochemistry for Spermidine and Glutamine Synthetase

The retinae from rats in the neonatal period (postnatal day 3: P3), in the weanling period (postnatal day 21: P21), and in early adulthood (postnatal day 120: P120) were analyzed for the expression of SPD. We used colocalization of SPD and GS expression to identify SPD localization in retinal Müller glial cells.

### 3.2. Glutamine Synthetase

**Figure 1:**
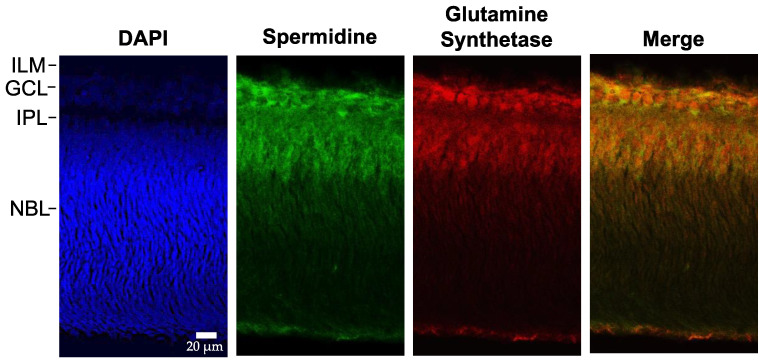
Spermidine (SPD) and Glutamine Synthetase (GS) immunolabeling in a 3-day-old (newborn: P3) rat retina. DAPI (blue) is a nuclear stain. SPD (green) is expressed in the neuroblast layer (NBL) surrounding cell nuclei and within the soma of some cells in the outermost part of the NBL. The SPD expression in these cells is co-localized with glial marker GS (red). Co-localized staining (merged, yellow). SPD is also expressed in the inner plexiform layer and in the ganglion cell layer, surrounding the cells. (GCL—Ganglion Cell Layer; IPL—Inner Plexiform Layer; NBL—Neuroblast Layer, proliferative zone of the inner optic cup that consists of retinal progenitor cells).

**Figure 2:**
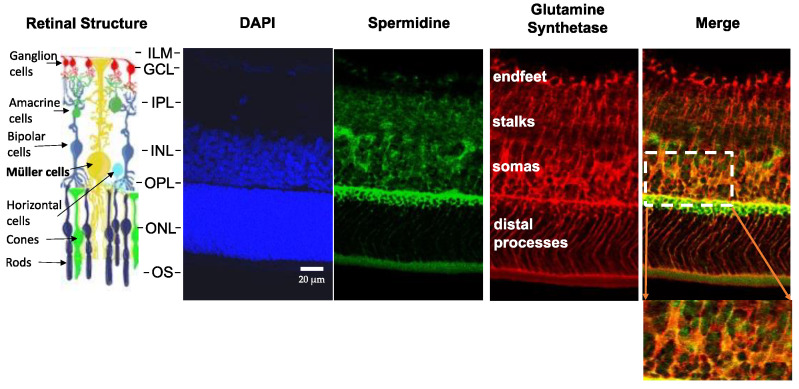
Spermidine (SPD) and Glutamine Synthetase (GS) immunolabeling in a 21-day-old (weanling: P21) rat retina. The retinal structure is represented by the schematic (left side) showing various types of retinal cells and the retinal layers. DAPI (blue) is a nuclear stain. SPD (green) is localized in Müller cell compartments and co-localized with the glial cell marker GS (red). Co-localized staining (merged, yellow). The strongest co-localization of SPD and GS was found in Müller cells (soma, endfeet, stalks, and distal processes) and in the area of contact of Müller cells with synapses of photoreceptors and their inner segments. Insert shows strong SPD labeling within the soma of Müller cells and their processes. (ILM-inner limiting membrane where endfeet of Müller cells make a border between the retina and vitreal humor; GCL-ganglion cell layer; IPL-inner plexiform layer, the synapses of neurons; INL-inner nuclear layer, the bodies of bipolar, horizontal cells and Müller cells; OPL-outer plexiform layer, the synapses of photoreceptors; ONL-outer nuclear layer, the cell bodies of photoreceptors; OS-outer segments-the photosensitive segments of photoreceptors).

**Figure 3:**
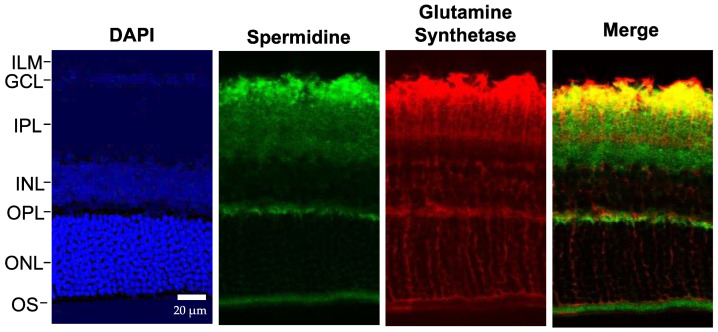
Spermidine (SPD) and Glutamine Synthetase (GS) immunolabeling in 120-day-old (young adult: P120) rat retina. DAPI (blue) is a nuclear stain. SPD (green) is localized in all Müller cell compartments and co-localized with glial cell marker GS (red). Co-localized staining (merged, yellow). If compared with P21 old retina (Figure 2) the SPD accumulation is less pronounced in soma of Müller cells of older rats but in endfeet the SPD label is strong. The pattern of co-localization (yellow) is similar in young rats (21 days old), specifically in the area of contact of photoreceptors with Müller cells (ILM-inner limiting membrane where endfeet of Müller cells make a border between the retina and vitreal humor; GCL-ganglion cell layer; IPL-inner plexiform layer, the synapses of neurons; INL-inner nuclear layer, the bodies of bipolar, horizontal cells and Müller cells; OPL-outer plexiform layer, the synapses of photoreceptors; ONL-outer nuclear layer, the cell bodies of photoreceptors; OS-outer segments-the photosensitive segments of photoreceptors).

We found robust expression of GS across all rat ages. In P3 rats, this marker was located across all layers and cell types (Figure 1), given that most cells at this age are undifferentiated progenitor cells. At this age, two main cell layers are identified, the ganglion cell layer and a neuroblast layer divided by an inner plexiform layer. The outer nuclear layer is yet not separated from the inner nuclear layer. In P21 rats and P120 rats, GS immunoreactivity was observed in all Müller cell compartments including the somata and distal processes with strong expression on the INL (inner nuclear layer) where the Müller cell somata are located, the synapses on the OPL (outer plexiform layer) and on the OS (outer segment) section of photoreceptors (Figure 2 and Figure 3).

### 3.3. Expression of the Polyamine Spermidine (SPD)

During early development (P3 rats), SPD immunolabel was found in the ganglion cell layer (GCL), in the inner part of the neuroblast layer (NBL) which is the closest to the inner plexiform layer (IPL), and within the processes of the radial Müller glia extending along the NBL (Figure 1). In the merged image of SPD with GS, SPD was observed co-localized with GS in the processes of the radial Müller glia in the NBL and surrounding the nuclei of cells in the inner part of the NBL and in the GCL.

In P21 rats, SPD was localized in all Müller cell compartments and co-localized with the glial cell marker GS (Figure 2). SPD was found in Müller cells (soma, endfeet, stalks, and distal processes) and in the area of contact between Müller cells and the photoreceptor cell nuclei (outer plexiform layer), as well as in the photoreceptor inner segment area. The strongest co-localization of SPD with GS was found in the somatic area of Müller cells, the distal processes of Müller cells that surround the nuclei in the ONL, and in the inner segment area of photoreceptors.

In P120 rats, SPD expression was concentrated in the inner limiting membrane area (ILM) which contains Müller cell endfeet, the outer plexiform layer (synaptic area), and the inner segment area of photoreceptors (Figure 3). At this age, the expression of SPD in the Müller cell somata and in the distal processes has strongly diminished. Prominent SPD co-localization with GS was observed in the ILM and Müller cell endfeet area.

**Figure 4:**
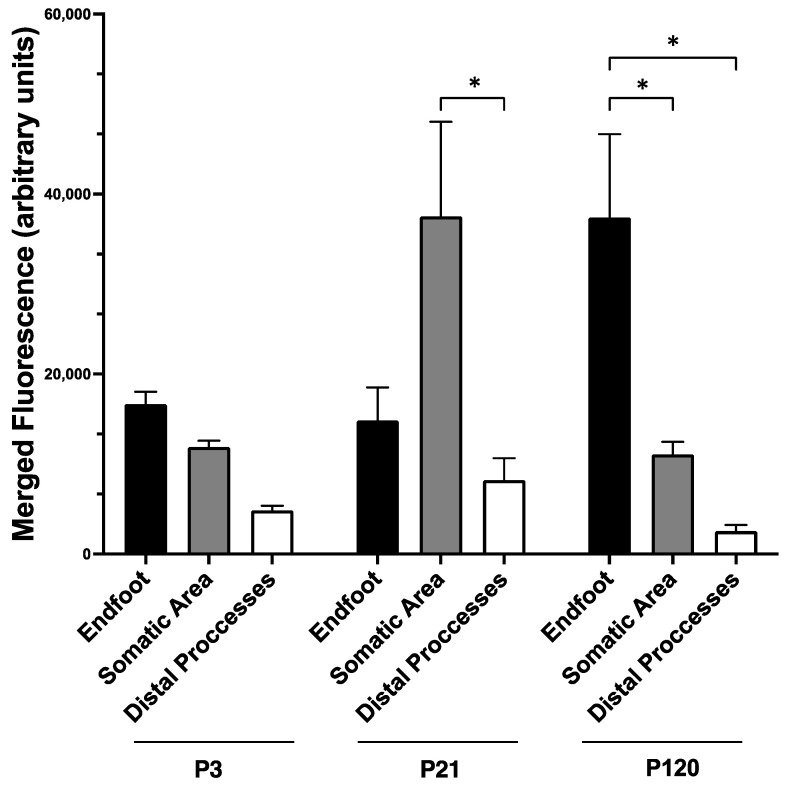
Co-localization of spermidine (SPD) and glutamine synthetase (GS) immunolabeling (merged fluorescence) in retinal layers of 3, 21, and 120 day old rats. In the area of Müller glial cell endfoot (black columns in all panels), SPD label is mostly co-localized with GS at the adult age of 120 days (right panel, black column). In the inner nuclear layer, a dramatic drop in the co-localization of SPD and GS occurs at P120 (right panel, gray column). Almost a complete lack of co-localization of SPD and the glial marker GS is seen in the outer nuclear layer by P120 (right panel, white column). Values with an asterisk (*) indicate *p* values lower than 0.05 with a 95% confidence interval which was considered statistically different. In the graph, the statistical difference within a group (same day) is shown. In addition, there were statistical differences amongst groups: endfoot (P3 vs. P120 and P21 vs. P120), somatic area (P3 vs. P21 and P21 vs. P120). Distal processes did not present any statistical difference. The data clearly show the shift of SPD label from multiple processes of Müller cells at a young age to the endfoot and synaptic areas (inner plexiform layer (IPL), outer plexiform layer (OPL)) in adults.

Figure 4 shows a semi-quantitative analysis of the staining intensity of SPD and GS by analyzing merged images using Image J software. We measured five fluorescence spots in every image in three different regions of the retina, (i) the endfoot area (corresponding to Müller cell terminal processes opposed to the vitreal body), (ii) the inner nuclear layer (corresponding to somata of bipolar, horizontal and Müller cells), and (iii) the outer nuclear layer (corresponding to photoreceptor (rods and cones) somata). Therefore, the analysis of co-localization of SPD and GS immunolabeling was performed in all principal retinal layers of P3, P21, and P120 rats. In the area of Müller glial cell endfeet, the SPD label is mostly co-localized with GS in the P120 retina (right panel, black column, Figure 4). In the inner nuclear layer, a dramatic drop in co-localization occurs at P120 (right panel, grey column, Figure 4). An almost complete lack of co-localization of SPD and glial marker GS was seen in the outer nuclear layer by P120 (right panel, white column, Figure 4). The data show clearly the shift of the SPD label from multiple processes of Müller cells at a young age to the endfoot and synaptic areas in adults.

## 4. Discussion

PAs are essential polycations for the stability of the CNS, specifically SPD [8,39,40]. PAs help to regulate known neuronal receptors and channels, such as ionotropic glutamate receptors including NMDA [15], AMPA/kainate [16], the inward rectifier Kir K^+^ channels [18,19,20,29,41,42], retinal cGMP-gated channels [31], acid-sensitive ionic channels (ASIC) [24], transient potential receptor channels (TRP) [22], and calcium-activated chloride channels [21]. In the retina, PAs and Kir4.1 channels are particularly essential for retinal pigment epithelial cell migration, to prevent glutamate-induced neurotoxicity, and for potassium homeostasis [41,42]. They are also neuroprotective for retinal ganglion cells [28] and decrease oxidative stress in pathological conditions such as traumatic optic neuropathy and glaucoma. Regardless of the necessity for PAs to brain [8,14,38] and retinal [19,27,31,32,36,37] balance, their content is known to markedly decrease with age in different tissues [38].

With aging, SPD content can be linked to multiple diseases such as Parkinson’s [43], Alzheimer’s [44], Huntington’s [45], multiple sclerosis [46], ALS [47], and syndromes such as EAST/SeSAME, Down, Rett where PAs play a key role [26,34,48]. These disorders are correlated with the dysfunction of PA turnover, and a treatment with SPD reduced oxidative stress and inflammation [13,26,34,44,45]. Unmodified PAs show striking modulatory actions such as anti-inflammatory, antioxidant [45,49], antidepressant, neuroprotective, and other beneficial effects including anti-microglial action and increasing longevity in vivo [50] as previously reviewed [7,8,15,26,48]. Daily intake of SPD was found to reduce ganglion cell death and enhance optic nerve regeneration following an optic nerve injury [28].

In the retina, defects in PA metabolism have been discovered in inherited disorders [51]. As discussed, PA content must be maintained to sustain the proper function of photoreceptors, retinal pigment epithelium, and Müller cells. Still, little is known about the precise localization, synthesis, and mechanisms of PA changes with age in the vertebrate retina. Therefore, given the importance of the presence of unmodified PAs as anti-inflammatory, antioxidant, and neuroprotective molecules [7,26], we were interested in understanding the precise localization of SPD (and their changes) with age in the rat retina. Previous studies by Ientile in 1986 [3] utilized rat retina to evaluate changes in PA content and biosynthesis with age and with induced toxicity by iodoacetate and sodium glutamate. First, SPM was at its highest concentration on postnatal day 12 and then there were drastic reductions in the concentrations of PUT SPD and SPM after postnatal day 16 [52]. Intriguingly, a remarkable depletion in SPM together with lower ODC and SAM-dc activities after gliotoxin iodoacetate was associated with loss of the rods and cones [3]. The authors used HPLC to determine PUT, SPD, and SPM content from retinal tissue and did not investigate specific cell types or where these PAs were localized.

Our results on the whole retina showed that during early development, progenitor radial glia cells widely express SPD. This could be attributed to their importance for the development and neuronal migration of neurons, photoreceptors, glia, and possibly, other cells (astrocytes, endothelial cells, retinal pigment epithelium, etc.). Similar results were observed in rabbit retina, where the concentration of PAs was found to decrease with age in photoreceptors (rods and cones) [36] and in fractioned rat retinae [3], where concentrations of spermine increased during development followed by a decrease after postnatal day 16. PA depletion caused the degeneration of photoreceptor cells [36]. Unfortunately, these authors do not report valuable information about Müller cells and age-dependent SPD expression in these cells.

Our results of P21 and P120 rat retina show that once cells have differentiated, Müller glia accumulate SPD. This accumulation is probably due to uptake mechanisms as was shown for astrocytes [10]. In cases of different PA-dependent diseases, the dietary intake of PAs such as agmatine and SPD can provide neuroprotection and life prolongation [50,53,54]. Nishimura in 2006 [38], showed that PAs decreased significantly in mouse thymus, spleen, ovary, liver, stomach, lung, kidney, heart, and muscle, but are stable in the brain. This suggests that glial cells accumulate PAs [27,33] by selective uptake [8,10,55].

Nishimura in 2006 and Soda in 2013 [38,56] suggested several sources of food enriched with PAs: pond smelt, turban shell viscera, salmon roe, cod roe, mushrooms, aged cheese, wheat germ, nuts, and many fermented products such as soybean (natto), pickles, beer, and wine that contain a large amount of PAs. Finally, Schwarz with co-authors in 2020 [57] showed that SPD dietary supplements and adherence to the Mediterranean diet increased the cortical and hippocampal mass of human volunteers compared with the control group. Taking into account that glial cells but not neurons accumulate PAs [8,19,27,33,37,48] and astrocytes take up SPD [10,55] it seems likely that the glial cells can be responsible for cognitive improvement. SPD nutrient supplementation has already shown very beneficial results against retinal ganglion cell degeneration [28]. In the future, these results may be translated to studies in humans to test the potential therapeutic benefit of nutrient supplementation with SPD to treat retinopathies, particularly in the aging population.

## 5. Conclusions

Since SPD immunostaining showed strong co-localization with the glial marker GS in Müller Cells (MCs) during all developmental and aging periods, we conclude that these glial cells accumulate SPD. Most interesting is the finding that SPD is translocated inside the glial cell compartments during aging. Initially, it is diffusely localized within MCs and ultimately resides in the endfoot processes and stalks. In addition, a near-complete decline of SPD content was observed in neurons with age.

## Data Availability

Additional data supporting the conclusions of this finding will be made available without undue reservation.
